# Expression of p53 predicts risk of prevalent and incident advanced neoplasia in patients with Barrett's esophagus and epithelial changes indefinite for dysplasia

**DOI:** 10.1093/gastro/gov045

**Published:** 2015-10-19

**Authors:** Bela Horvath, Prabhdeep Singh, Hao Xie, Prashanthi N Thota, Xingwen Sun, Xiuli Liu

**Affiliations:** ^1^Department of Anatomic Pathology, The Cleveland Clinic, Cleveland, OH, USA; ^2^Department of Gastroenterology, Digestive Disease Institute, The Cleveland Clinic, Cleveland, OH, USA; ^3^Department of Internal Medicine, Yale University School of Medicine, New Haven, CT, USA; ^4^Univeristätsmedizin, Georg-August-University of Göttingen, Göttingen, Germany

**Keywords:** Barrett’s esophagus, dysplasia, esophageal adenocarcinoma

## Abstract

**Background and aims:** Patients with Barrett’s esophagus (BE) are at an increased risk for developing esophageal adenocarcinoma (EAC); thus they may undergo regular endoscopic surveillance. If epithelial changes cannot be unequivocally classified as negative or positive for dysplasia, a diagnosis of indefinite for dysplasia (IND) is recommended. Several biomarkers have been proposed as markers or predictors of neoplasia in the general BE population; however, their significance is not clear in patients with BE-IND. We therefore performed a retrospective study to determine whether expression of these biomarkers was associated with the development of neoplasia in BE-IND patients.

**Methods:** We searched our archives to identify all cases of BE-IND diagnosed between January 1992 and December 2007. Immunohistochemical analyses were used to semi-quantify the expression of p53, α-methylacyl-CoA racemase (AMACR), and cyclin D1. A univariate analysis was used to identify predictors for prevalent and incident neoplasia and advanced neoplasia.

**Results:** Among the 103 patients with an index diagnosis of BE-IND who were included in this study, 81 (78.6%) underwent a follow-up biopsy within 12 months of diagnosis; 10 (12.3%) had neoplasia, including four (4.9%) with advanced neoplasia. Among 79 patients without prevalent neoplasia who underwent more than 1 year of follow-up, 18 (22.8%) had developed neoplasia, including four (5.1%) with advanced neoplasia. AMACR and cyclin D1 expression levels were not correlated with prevalent or incident neoplasia; however, high p53 expression (>5%) was associated with prevalent advanced neoplasia on surveillance biopsy (*P* = 0.04) and with an increased risk of progression to advanced neoplasia (HR = 12; *P* = 0.03).

**Conclusion:** In this study, p53 expression was found to be predictive of prevalent advanced neoplasia and progression to advanced neoplasia in patients with BE-IND.

## Introduction

Patients with Barrett’s esophagus (BE) are at a higher risk of experiencing neoplastic progression to esophageal adenocarcinoma (EAC) than the general population [1–3]. Thus, endoscopic surveillance guidelines have been established for detecting early dysplasia and EAC in these patients [4, 5]. Surveillance intervals are based on the degree of dysplasia, as it has been found to be the best predictor of neoplastic progression [6, 7].

Esophageal dysplasia is defined as unequivocal neoplastic alteration in the epithelium [8]. Biopsy samples of BE are classified as negative for dysplasia, positive for dysplasia [including low-grade (LGD) and high-grade dysplasia (HGD)], or indefinite for dysplasia (IND) [9]. A neoplastic lesion is defined as advanced if it contains an area of HGD and/or carcinoma. A biopsy sample is classified as IND if the epithelial changes are insufficient to establish beyond doubt the presence or absence of dysplasia or if other conditions preclude an unequivocal diagnosis (such as extensive inflammation) [10].

We previously demonstrated that there is a significant risk of prevalent and incident neoplasia in patients with BE-IND [11]; multifocality of epithelial IND changes and longer segment of BE are associated with neoplastic progression. Recently, several biomarkers have been proposed as markers or predictors of neoplasia in the general BE population [12–18]; however, the significance of these biomarkers is not clear in patients with BE-IND, a histologically challenging diagnosis.

In this retrospective study, we determined the association between the biomarkers p53, alpha-methylacyl-CoA racemase (AMACR), and cyclin D1 and the development of prevalent neoplasia and incident neoplasia in patients with BE-IND.

## Patients and methods

Departmental databases at the Department of Pathology of the Cleveland Clinic were searched to identify cases of BE-IND diagnosed from January 1992 to December 2007. Cases were excluded if (i) there was a previous or synchronous diagnosis of definitive neoplasia (LGD, HGD, or EAC), (ii) if medical charts or slides were missing, (iii) if there was inadequate tissue for immunohistochemical evaluation or (iv) if they were lost to follow-up. During endoscopic surveillance of BE, the Barrett’s segment was biopsied at four quadrants at 2 cm intervals. Areas with suspected or known dysplasia were biopsied at 1 cm intervals. Biopsy specimens of nodules or ulcers were submitted separately.

Patient demographics, clinical parameters, and pathological information were extracted from a chart review. Prevalent neoplasia was defined as the presence of LGD or advanced neoplasia (HGD or EAC) on surveillance biopsy or tumor resection within 12 months following the diagnosis of IND. Neoplastic changes beyond this period were defined as incident neoplasia.

A histological review of the slides was performed as described previously [11]; in brief, five gastrointestinal pathologists reviewed the slides in a blinded fashion. Epithelial changes were classified as negative for dysplasia, positive for dysplasia (LGD, HGD, or EAC) or IND. Immunohistochemical staining for p53, AMACR, and cyclin D1 was performed on whole tissue sections from Hollande's fixed or formalin-fixed and paraffin-embedded tissue, as described previously [19]. In brief, de-paraffinized tissue sections were stained with antibodies against p53 (clone DO-7, at a 1:20 working dilution; Dako Corp., Carpinteria, CA, USA), AMACR (clone 13H4, at a 1:100 working dilution, Zeta Corp., Sierra Madre, CA, USA), and cyclin D1 (clone SP4, at a 1:100 working dilution; ThermoLabVision, Waltham, MA, USA). We used diaminobenzidine as the chromogen. Appropriate positive and negative controls were reviewed.

**Figure 1.**
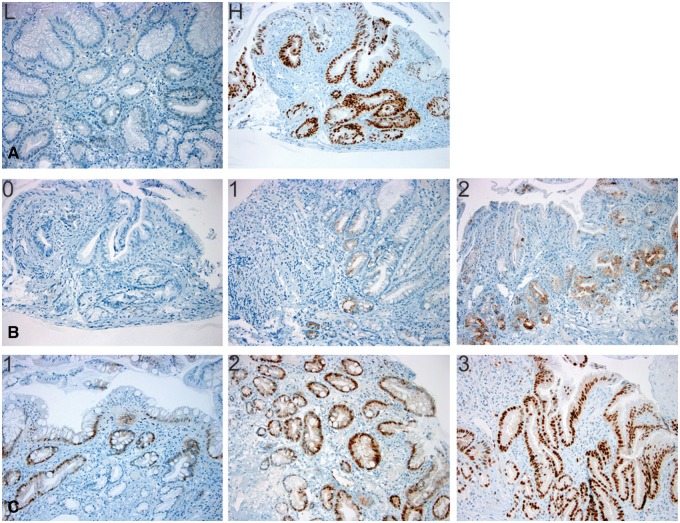
Examples of immunohistochemical staining for p53, AMACR, and cyclin D1 in BE-IND biopsy samples. The presence or absence of nuclear p53 staining was evaluated in esophageal columnar tissue. Its expression was determined as a percentage of epithelial cells showing nuclear staining within a high-power field (**A:** peroxidase stain, ×200, low ≤5%, high >5%). Cytoplasmic AMACR staining was graded as 0 (no visible staining), 1+ (any identifiable staining), or 2+ (widespread strong staining) (**B:** peroxidase staining, ×200). Nuclear cyclin D1 staining was graded as 0 (no visible staining), 1+ (any identifiable staining), 2+ (widespread strong staining), or 3+ (widespread intense staining) (**C:** immunoperoxidase staining, ×200).

All immunohistochemical stains were evaluated by one pathologist (X.L.) who was blinded to the neoplasia outcomes. The expression of p53 was determined as the percentage of epithelial cells in the esophageal columnar tissue showing nuclear staining within a high-power field [19] (Figure 1A). High p53 expression was defined as more than 5% of the epithelial cells in the esophageal columnar tissue showing nuclear staining. Nuclear cyclin D1 and cytoplasmic AMACR staining was graded as 0 (no visible staining), 1+ (any identifiable staining), 2+ (widespread strong staining) or 3+ (widespread intense staining), as previously described [17] (Figures 1B and 1C).

**Figure 2.**
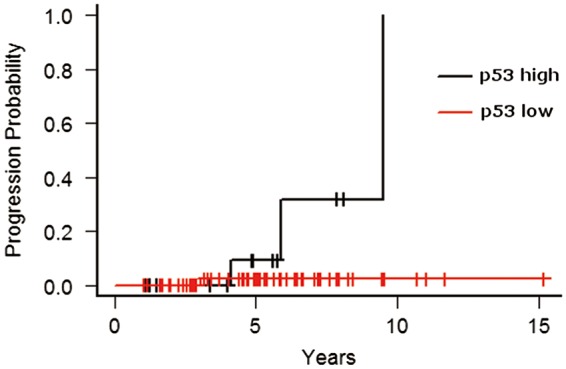
High expression of p53 in the index BE-IND biopsy sample was associated with progression to advanced neoplasia (*P* = 0.03).

Continuous variables were compared using Wilcoxon rank-sum test. Categorical variables were compared using Fisher’s exact test. The rates of progression to neoplasia (LGD, HGD, or EAC) and advanced neoplasia (HGD or EAC) were determined in the incident neoplasia group. Patients without LGD, HGD, or EAC during follow-up were censored at their last endoscopic surveillance. The Kaplan-Meier method was used to estimate the probability of progression, which was compared using the log-rank test. A Cox proportional hazards model was used for the univariate analysis. Statistical significance was defined as a *P-*value of <0.05.

## Results

We identified 225 patients with a diagnosis of BE-IND. After an extensive review of the medical records, we excluded 122 due to (i) a previous or synchronous diagnosis of definitive neoplasia (LGD, HGD, or EAC) (*n* = 70), (ii) being lost to follow-up (*n* = 37), (iii) lacking slides or BE (*n* = 11) or (iv) not having enough tissue for immunohistochemical evaluation (*n* = 4). Thus, our final patient population was 103. No cases were collectively diagnosed as dysplasia by the reviewing pathologists.

### Predicting prevalent neoplasia in patients with BE-IND

Eighty-one of the 103 patients (78.6%) underwent a surveillance biopsy within 12 months of the IND diagnosis; 10 (12.3%) were found to have neoplasia, with a 4.9% prevalence of advanced neoplasia (two with HGD and two with EAC).

There was no association between clinico-demographic parameters, endoscopic parameters, or AMACR or cyclin D1 expression levels and the presence of prevalent neoplasia or advanced prevalent neoplasia (Tables 1 and 2 and data not shown); high p53 expression (>5%), however, was associated with the presence of advanced prevalent neoplasia (Table 2).

**Table 1.** Association between biomarkers and prevalent neoplasia in patients with BE-IND

Biomarker	Prevalent neoplasia	No prevalent neoplasia	*P*-value
p53 (≤5%/>5%)	6/4	55/16	0.3
AMACR (0/1/2)	2/6/2	15/40/16	1
Cyclin D1 (0/1/2/3)	0/1/8/1	1/4/53/13	0.7

BE = Barrett’s esophagus; IND = indefinite for dysplasia

**Table 2.** Association between demographic and clinical parameters and biomarkers and advanced prevalent neoplasia in patients with BE-IND

Parameter	Advanced prevalent neoplasia	No advanced prevalent neoplasia	*P*-value
Sex (female/male)	0/4	21/56	0.6
Family history of EAC (yes/no)	0/3	1/72	1
Duration of BE ( <10/≥10 years)	0/3	25/47	0.5
BE irregularity (yes/no)	2/2	14/54	0.2
Esophagitis (yes/no)	1/2	11/58	0.4
PPI use (yes/no)	2/2	57/15	0.2
NSAID use (yes/no)	0/4	5/68	1
Aspirin use (yes/no)	1/3	12/60	0.5
Smoking (current/former/never)	0/4/0	8/34/31	0.2
Alcohol abuse (current/former/never)	2/0/2	32/3/38	1
p53 (>5%/≤5%)	3/1	17/60	**0.045****^a^**
AMACR (0/1/2)	1/2/1	16/44/17	1
Cyclin D1 (0/1/2/3)	0/0/4/0	1/5/57/14	1

^a^Bold font indicates statistical significance for *P*-value <0.05

BE = Barrett’s esophagus; EAC = esophageal adenocarcinoma; IND = indefinite for dysplasia; NSAID = non-steroidal anti-inflammatory drug; PPI = proton pump inhibitor

### Predicting incident neoplasia in patients with BE-IND

Among the 79 patients who were proven or presumed not to have prevalent neoplasia but more than 1 year of follow-up [median: 59 months (range: 13–182 months)], 18 (22.8%) developed incident neoplasia, including four with advanced neoplasia (two with HGD diagnosed as 9.5 and 5.9 years after BE-IND and two with EAC diagnosed at 4.1 and 3.0 years after BE-IND). The case that developed incident HGD at 9.5 years after BE-IND had no endoscopic examination within 1 year following BE-IND; the remaining three cases had no prevalent neoplasia by endoscopic surveillance within 1 year after BE-IND.

An analysis of clinico-demographic and endoscopic parameters confirmed that only BE length was correlated with neoplastic progression (*P* = 0.02; data not shown). Patient age, sex, hiatal hernia length, body mass index (BMI), family history of EAC, esophagitis or BE mucosal irregularities (as determined by endoscopy), aspirin or NSAID use, and current or former smoking or alcohol abuse, AMACR and cyclin D1 expression levels were not associated with neoplastic progression or advanced neoplastic progression (Tables 3 and 4, and data not shown); however, high p53 expression (>5%) was associated with progression to advanced neoplasia on univariate analysis (hazard ratio 12; *P* = 0.03) (Table 5).

**Table 3.** Association between biomarkers and incident neoplasia in patients with BE-IND

Biomarker	Incident neoplasia	No incident neoplasia	*P*-value
p53 (>5%/≤5%)	5/13	14/47	0.8
AMACR (0/1/2)	2/12/4	15/29/17	0.4
Cyclin D1 (1/2/3)	2/12/4	3/52/6	0.2

BE = Barrett’s esophagus; IND = indefinite for dysplasia

**Table 4.** Association between demographic and clinical parameters and biomarkers and the development of advanced incident neoplasia in patients with BE-IND

Parameter	Incident neoplasia	No incident neoplasia	*P*-value
Age (years)	60.5 (58–72)	63 (30–85)	0.8
Number of endoscopic follow-ups	2 (0–4)	2 (0–9)	0.7
Follow-up duration (years)	5.0 (3.0–9.5)	5 (1.1–15.2)	1
Length of BE (cm)	6 (6–6)	4 (0–15)	1
Length of hiatal hernia (cm)	3.5 (3–4)	3 (0–12)	1
Body mass index (kg/m^2^)	25.9 (21–33)	28.9 (18–47)	0.4
Sex (female/male)	0/4	18/57	0.6
Family history of EAC (yes/no)	0/4	2/68	1
Duration of BE (<10/≥10 years)	0/3	23/47	0.5
BE irregularity (yes/no)	1/1	13/54	0.4
Esophagitis (yes/no)	0/2	11/57	1
PPI use (yes/no)	3/0	51/17	1
NSAID use (yes/no)	0/3	7/62	1
Aspirin use (yes/no)	0/3	14/54	1
Smoking (current/former/never)	0/4/0	7/30/31	0.2
Alcohol abuse (current/former/never)	2/0/2	32/2/34	1
p53 (>5%/≤5%)	3/1	16/59	**0.04**^a^
AMACR (0/1/2)	1/2/1	16/39/20	1
Cyclin D1 (1/2/3)	1/3/0	4/61/10	0.3

Continuous values were presented as medians (range).

^a^Bold font indicates statistical significance for *P*-value <0.05.

BE = Barrett’s esophagus; EAC = esophageal adenocarcinoma; IND = indefinite for dysplasia; NSAID = non-steroidal anti-inflammatory drug; PPI = proton pump inhibitor

**Table 5.** Univariate analysis of risk parameters for the development of advanced incident neoplasia in patients with BE-IND

**Risk parameter**	**Incidence rate (95% CI), %**	**Hazard ratio (95% CI)**	***P*-value**
Age (years)	^a^	1.03 (0.95–1.1)	0.5
Number of endoscopic follow-ups	^a^	0.8 (0.5–1.3)	0.3
BE length (cm)	^a^	1.05 (0.6–1.7)	0.8
Hiatal hernia length (cm)	^a^	0.97 (0.5–1.8)	0.9
BMI (kg/m^2^)	^a^	0.93 (0.8–1.1)	0.5
Sex (male/female)	^a^	^a^	^a^
BE duration (≥10/<10 years)	^a^	^a^	^a^
BE irregularity (yes/no)	1.4 (0.2-10)/0.3 (0.05-2.4)	4.1 (0.3–65)	0.3
Esophagitis (yes/no)	^a^	^a^	^a^
PPI use (yes/no)	^a^	^a^	^a^
NSAID use (yes/no)	^a^	^a^	^a^
Aspirin use (yes/no)	^a^	^a^	^a^
Smoking (former + current/never)	^a^	^a^	^a^
Alcohol abuse (former + current/never)	1.0 (0.2-3.9)/1.1 (0.3-4.4)	0.7 (0.1–5.1)	0.7
p53 expression (>5%/≤5%)	3.5 (1.1-11)/0.3 (0.04-2.2)	12 (1.43–100)	**0.03**^b^
AMACR expression (2/1, 0)	1.1 (0.2-7.9)/0.9 (0.3-2.9)	2.3 (0.2–31)	0.5
Cyclin D1 expression (3, 2/1)	0.8 (0.2-2.4)/4.9 (0.7-35)	0.1 (0.01–1.1)	0.06

^a^Values cannot be calculated due to small sample size.

^b^Bold font indicates statistical significance for *P*-value <0.05.

BE = Barrett’s esophagus; CI = confidence interval; EAC = esophageal adenocarcinoma; IND = indefinite for dysplasia; NSAID = non-steroidal anti-inflammatory drug; PPI = proton pump inhibitor

## Discussion

Tumor protein p53 is involved in the regulation of the cell cycle and functions as a tumor suppressor [20]. Several previous studies have demonstrated that p53 deactivation has a role in esophageal carcinogenesis [12, 21, 22]. Due to the greater half-life of mutant p53, the protein accumulates and its nuclear expression can be assessed by immunohistochemical analysis [23]. In the present study we showed, for the first time, that high p53 expression in an index BE-IND biopsy sample is associated with both prevalent and incident advanced neoplasia on subsequent surveillance biopsies.

AMACR, a metabolic enzyme that is involved in the metabolism of branched-chain fatty acids [24], is now a widely used marker in the interpretation of prostate biopsy samples. It has been shown to play a role in the pathogenesis of gastrointestinal malignancies, including colorectal and esophageal adenocarcinoma [13, 25, 26]. The results of a recent paper indicated that strong AMACR expression has a slightly higher positive predictive value than does LGD for advanced neoplasia in the general BE population [14]. In our BE-IND patient cohort, however, we did not show any correlation between AMACR expression and prevalent or incident neoplasia. This observation contradicts the results of a smaller study of 37 cases of BE-IND [27], in which AMACR expression had a positive predictive value of 0.44 and negative predictive value of 0.92 for neoplastic progression. These contradictory results might be accounted for by (i) the low inter-observer agreement of the diagnosis [11], (ii) different antibodies or scoring criteria or (iii) a different composition or size of the investigated patient population.

Cyclin D1, similarly to p53, plays a key role in regulating the transition from G1 to S phase [28]. Increased expression of the cyclin D1 gene was found in both dysplastic and non-dysplastic BE and may be an early event in the tumorigenic process of EACs [18]. While some authors have found a correlation between cyclin D1 overexpression and the degree of dysplasia or the likelihood of progression [16–18], others were not able to reproduce these findings [29, 30]. In our cohort, we found almost universal expression of cyclin D1 [confirming its early expression in carcinogenesis (18)], but we were unable to show an association between cyclin D1 expression and neoplastic progression.

Our study is one of the most comprehensive analyses of a BE-IND patient population to date but it has certain limitations. We were unable to perform a multivariate analysis due to the sample size; in addition, it was a retrospective analysis, which could have affected the quality of our data. As we obtained data from a highly specialized tertiary care center, referral bias may have been present in our study.

In summary, we showed that p53 immunohistochemical analysis is a valuable predictor of advanced neoplasia—both prevalent and incident—in patients with BE-IND, a heterogeneous group in whom clinico-demographic parameters often fail as a risk stratification tool. Additional studies are needed to confirm the predictive value of p53 in BE-IND, to aid patient selection for endoscopic surveillance programs.

*Conflict of interest statement*: none declared.
